# Microbiome Profiles in Periodontitis in Relation to Host and Disease Characteristics

**DOI:** 10.1371/journal.pone.0127077

**Published:** 2015-05-18

**Authors:** Bo-Young Hong, Michel V. Furtado Araujo, Linda D. Strausbaugh, Evimaria Terzi, Effie Ioannidou, Patricia I. Diaz

**Affiliations:** 1 Division of Periodontology, Department of Oral Health and Diagnostic Sciences, The University of Connecticut Health Center, Farmington, Connecticut, United States of America; 2 The Center for Applied Genetics and Technologies, Department of Molecular and Cell Biology, The University of Connecticut, Storrs, Connecticut, United States of America; 3 Department of Computer Science, Boston University, Boston, Massachusetts, United States of America; University of Florida, UNITED STATES

## Abstract

Periodontitis is an inflammatory condition that affects the supporting tissues surrounding teeth. The occurrence of periodontitis is associated with shifts in the structure of the communities that inhabit the gingival sulcus. Although great inter-subject variability in the subgingival microbiome has been observed in subjects with periodontitis, it is unclear whether distinct community types exist and if differences in microbial signatures correlate with host characteristics or with the variable clinical presentations of periodontitis. Therefore, in this study we explored the existence of different community types in periodontitis and their relationship with host demographic, medical and disease-related clinical characteristics. Clustering analyses of microbial abundance profiles suggested two types of communities (A and B) existed in the 34 subjects with periodontitis evaluated. Type B communities harbored greater proportions of certain periodontitis-associated taxa, including species historically associated with the disease, such as *Porphyromonas gingivalis*, *Tannerella forsythia* and *Treponema denticola*, and taxa recently linked to periodontitis. In contrast, subjects with type A communities had increased proportions of different periodontitis-associated species, and were also enriched for health-associated species and core taxa (those equally prevalent in health and periodontitis). Periodontitis subgingival clusters were not associated with demographic, medical or disease-specific clinical parameters other than periodontitis extent (proportion of sites affected), which positively correlated with the total proportion of cluster B signature taxa. In conclusion, two types of microbial communities were detected in subjects with periodontitis. Host demographics and underlying medical conditions did not correlate with these profiles, which instead appeared to be related to periodontitis extent, with type B communities present in more widespread disease cases. The two identified periodontitis profiles may represent distinct dysbiotic processes potentially requiring community-tailored therapeutic interventions.

## Introduction

Studies of the microbial communities that exist at various human body compartments have detected great inter-subject variability in microbial abundances within sites, in some cases finding that patterns of abundance determine discrete community types across subjects [1–4]. Although there is lack of agreement regarding the best approaches to define community types, it is clear that even in the homogenous cohort of healthy subjects characterized via the Human Microbiome Project (HMP), patterns of variation consistent with discrete community types exist [1, 2]. Variability patterns in healthy populations could result from community assembly historic events and are likely influenced by a range of phenotypic characteristics, still within the boundaries of clinical health, present at host sites. Different community types in health could affect susceptibility to disease and have therefore been the focus of several studies [1–4]. In the meanwhile, variability among microbial communities associated with human diseases has received less attention. Subjects suffering from a disease usually constitute a less homogeneous population since apart from uneven clinical manifestations, they are also commonly affected by concomitant conditions and undergo diverse therapeutic interventions, all potentially modifying the microbial communities directly associated with their affecting condition. Defining variability in microbial communities in disease, however, is equally important as in health, particularly in the case of diseases of microbial etiology in which distinct community patterns could indicate a need for different therapeutic approaches against the same clinical entity.

Periodontitis is an inflammatory condition that affects the supporting structures of teeth with the microbial communities that inhabit the subgingival environment serving as the inflammatory trigger [5]. The development of periodontitis is associated with an increase in microbial load and dramatic shifts in community structure, in a process that resembles microbial succession, with primary health-associated species remaining part of periodontitis communities, albeit in low proportions, and a diverse range of periodontitis-associated taxa becoming numerically dominant as the shifts occur [6, 7]. Subjects with periodontitis are frequently smokers or suffer from diabetes [8–11]. Chronic kidney disease (CKD) has also been associated with increased periodontitis prevalence [12]. While smoking seems to act as a direct modifier of the oral flora [13], it is less clear if conditions such as diabetes or CKD, which affect immune responses, serve as modifiers of the subgingival microbiome. Similarly, periodontitis occurs in a wide range of subjects with diverse demographic characteristics showing increased prevalence in males [8]. The disease clinical presentation is also variable, with non-uniform patterns in terms of teeth and sites affected, severity of periodontal pockets and spread of the lesions, with little knowledge available on the relationship between microbial profiles and variability in periodontitis clinical presentations. Classic DNA-DNA checkerboard studies including healthy and periodontitis cohorts have shown that the presence, levels and proportions of certain species (mainly the red complex, *Porphyromonas gingivalis*, *Tannerella forsythia* and *Treponema denticola*) correlate with clinical indicators of periodontitis [14–16]. Few attempts, none using high throughput sequencing approaches, have been made to study the relationship between microbial profiles and clinical parameters within periodontitis cohorts [17–19], and only one study, via the DNA-DNA checkerboard hybridization technique, has evaluated the presence of different community types among subjects with periodontitis [20].

The goal of this study was to explore variability in microbiome profiles in a heterogeneous cohort of subjects suffering from periodontitis. A possible relationship among community types and differing host and disease characteristics was then explored using a “top down” approach. We identified, using clustering techniques, two community types (A and B) in subjects with periodontitis, although clusters had low cohesion and separation metrics. Community types did not correlate with demographic or medical characteristics of subjects. However, subjects with greater number of sites affected by periodontitis harbored type B communities, with the total abundance of type B signature taxa correlating with periodontitis extent indicators. We show that type B communities are enriched for the classic red complex periodontitis-associated species [15], apart from other less known periodontitis-associated taxa, while type A communities are enriched for different periodontitis-associated taxa, health-associated and core species. The two community types may represent different dysbiotic processes associated with periodontitis.

## Materials and Methods

### Studied subjects, data collection and sampling

Subjects with periodontitis were enrolled between 2010 and 2012 through clinical protocols approved by the Institutional Review Board from the University of Connecticut Health Center (UCHC), IRB numbers 10-009-2 and 11-139-3. All participants provided written informed consent in forms approved by the IRB Committee. Recruitment sites were the Periodontology Graduate Clinic at UCHC, the University of Connecticut Dialysis Center in Farmington, CT and the Springfield Dialysis Center in Springfield, MA. Therefore, some subjects had end-stage chronic kidney disease (CKD). Diabetics were also not excluded from the study. Inclusion criteria for subjects with periodontitis were: (1) at least one site with a pocket depth (PD) ≥ 5 mm and ≥ 2 interproximal sites with clinical attachment level (CAL) ≥ 6 mm (Page & Eke 2007), (2) a minimum of 15 teeth, (3) no history of smoking, (4) no antibiotic use within the last month, and (5) no periodontal treatment within one year. A one month antibiotic-free period prior to study enrollment was chosen to increase recruitment in this medically-heterogeneous population and was based on studies on gut communities, which show a rapid recovery of community composition after 4 weeks of antibiotic administration [21]. It should be noted, however, that there is lack of evidence on the optimal recovery period for oral communities after antibiotic use, with studies on gut communities suggesting that a complete community recovery does not occur even 6 months post-antibiotics [22]. Medical and demographic data were recorded and a comprehensive oral examination was performed combined with the collection of subgingival plaque from the deepest pocket in two different quadrants. The plaque sample collection procedure involved removal of supragingival plaque with a sterile curette, followed by insertion of a newly sterilized curette to the depth of the pocket and removal of the sample by a single curette stroke. Plaque samples from each subject were pooled in 50 μl Tris-EDTA buffer and stored at -80°C.

### DNA isolation, 16S rRNA gene library preparation and sequencing

DNA was isolated as previously described [23]. Amplicon libraries of 16S rRNA gene V1-V2 hypervariable regions were generated in triplicate using fusion primers, which included universal primers 8F 5’agagtttgatcmtggctcag3’ and 361R 5’cyiactgctgcctcccgtag3’ [24]. PCR and library preparation procedures have been described [23]. Libraries were sequenced in the forward direction using 454 Titanium chemistry. Sequences are available at the Short Reads Archive (SRP038001).

### Sequence data processing and analysis

Raw data files were processed in Mothur following a published pipeline [25, 26]. Briefly, datasets were denoised, trimmed, and chimeras removed, followed by OTU analysis, clustering sequences at 97% similarity, a threshold we have found allows discrimination of the majority of oral species when evaluated by high throughput sequencing of the V1-V2 hypervariable region. Individual sequences were classified using Mothur’s version of the Ribosomal Database Project (RDP) classifier [27] and the Human Oral Microbiome Database (HOMD) [28] as template. We have found that the use of the RDP classifier and the HOMD curated database allows correct species-level assignments for 90% of V1-V2 short reads of oral taxa in the HOMD, in close agreement with the use of full length 16S rRNA gene sequences (92% full-length sequences are correctly identified using the same strategy). As a control, we also used the broader RDP trainset as a taxonomy template to verify genus-level classifications. Each OTU was then assigned a taxonomy based on the majority of sequences within the OTU. When a consensus taxonomic assignment at the species level was not possible, the taxonomical rank closest to species with a consensus was reported. In this case, the representative sequence for each OTU (middle sequence) was also compared via BLAST to the HOMD and its taxonomy reported in parentheses if there was >97% similarity to a HOMD Oral Taxon (OT).

Hierarchical clustering of periodontitis communities based on OTU relative abundances was performed via complete linkage using Euclidean distances and the hclust function in R. For these analyses relative abundance values were transformed using the inverse hyperbolic sine method [29]. Heatmaps were created via the heatmap.2 function in R. Clustering was also performed using a *k*-means algorithm (*k* = 2). To test significance we computed the silhouette coefficient (SI) [30] of the hierarchical and k-means clusters, performed randomization tests creating 10,000 random clusters and calculated the SI for each random cluster, comparing it with the SI of the original clusters. Differences in subject parameters between clusters were tested with t-tests, Mann-Whitney U-tests or χ^2^ as appropriate. Correlations between relative abundances of individual OTUs or the sum of relative abundances of cluster A or B signature OTUs and clinical parameters were performed via Spearman rank order tests. The significance threshold for all statistical tests was adjusted using the Benjamini-Hochberg false discovery rate method.

## Results

### Two types of microbial communities, related to subject-level indicators of disease severity/extent, exist in periodontitis

**Fig 1:**
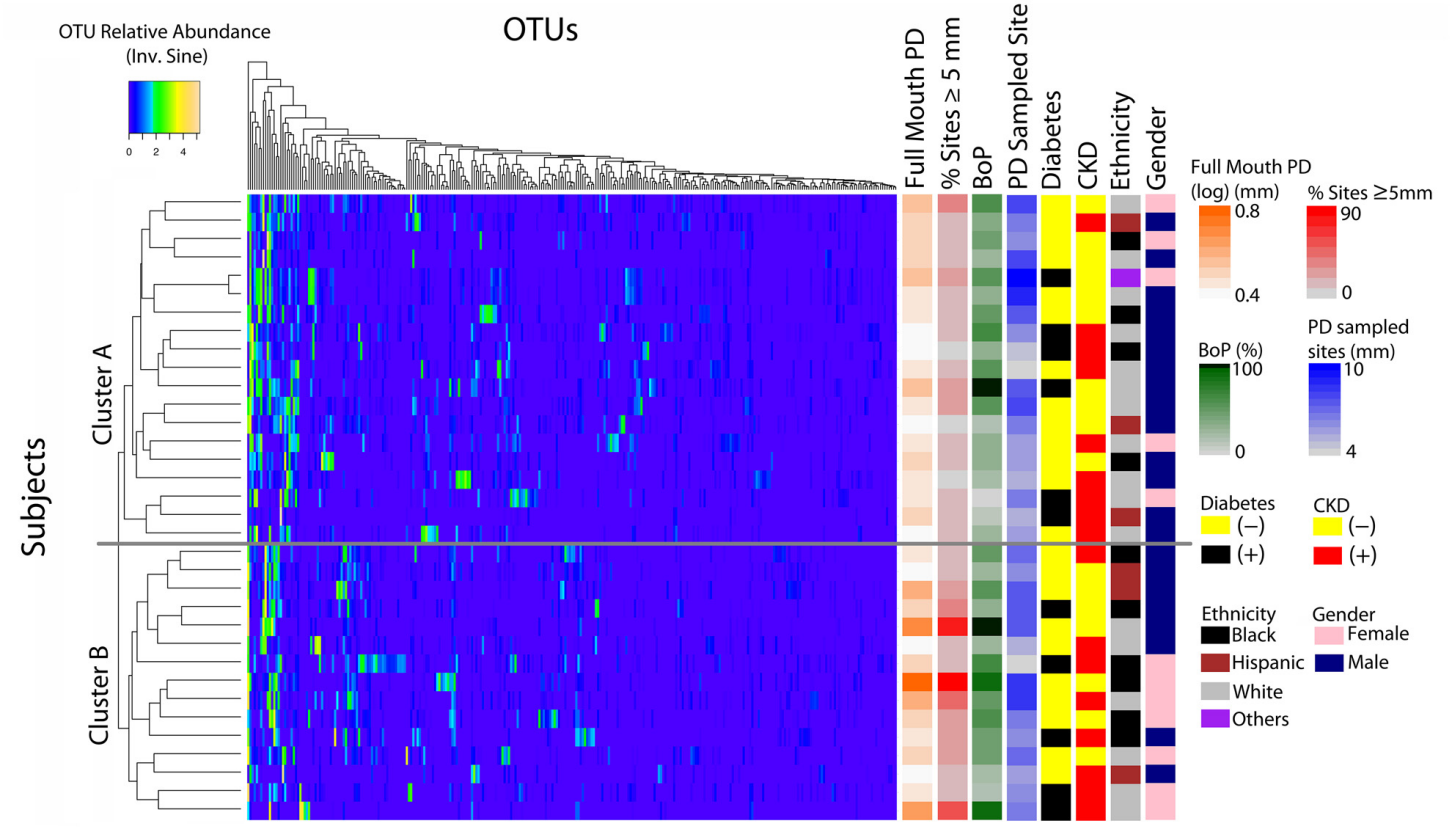
Inter-subject variability of subgingival communities in periodontitis and relationship of microbial profiles with demographic, medical and clinical characteristics. Graph shows a heatmap of the transformed relative abundances for the top 300 OTUs found in the subgingival microbial communities of subjects with periodontitis. Subjects are shown in rows, while OTUs appear in columns. Color scale for heatmap appears in the top left with the most abundant OTUs in yellow and the least abundant in blue. Subject raws are organized according to results of unsupervised hierarchical clustering (complete linkage) of microbial communities based on OTU relative abundances. Dendogram on the left shows a tendency for 2 clusters to form (A and B). Color bars in the right depict the demographic, medical and clinical characteristics for each subject. Color scales for metadata appear on the right side.

Table 1 shows the demographic, medical and clinical characteristics of the studied subjects. Seventeen subjects had CKD, of whom 8 were diabetic, while seventeen subjects did not have CKD with 3 diabetics in this subgroup (for a total of 11 diabetic subjects out of 34 total subjects). 16S rRNA gene sequence libraries were randomly subsampled to contain the same number of sequenced reads (n = 4,670). Among these subsampled libraries, we found 958 OTUs. Unsupervised hierarchical clustering of periodontitis samples based on OTU abundance revealed a tendency for samples to separate into two groups (A and B), depicted in Fig 1, with 19 subjects in cluster A and 15 subjects in cluster B. These groups were 100% concordant when clustering was performed with all OTUs found or with the top 300 OTUs (mean relative abundance >0.02%) (S1 Fig). For simplicity, later analyses were performed with the top 300 OTUs.

**Table 1:** Clinical and demographic characteristics of studied subjects with chronic periodontitis (n = 34).

Age (years)	52.0±12.0
Gender (% male)	64.7
Ethnicity (% non-whites)	44.1
Diabetes status (% yes)	29.4
CKD status (% yes)	50.0
PD (mm)	3.2 ±0.8
CAL (mm)	3.6 ±1.0
BoP (% of sites)	44.0 ±25.0
PS (% of sites)	66.0 ±23.0
PD ≥ 5mm (% of sites)	19.0 ±18.0
PD sampled sites (mm)	7.0 ±1.5
CAL sampled sites (mm)	8.0 ±2.1

PD: pocket depth; CAL: clinical attachment level; BoP: bleeding on probing; PS: plaque score. Data represent mean ± standard deviation or frequencies (%).

Clusters obtained via a k-means algorithm (16 subjects placed in cluster A and 18 subjects in cluster B) largely agreed with the hierarchical clusters, with only 14% of subjects falling into a different cluster (S2 Fig). Lack of 100% agreement suggested clusters were not strong. This was confirmed by their SI, which was 0.16 for the complete-linkage hierarchical clusters and 0.19 for the *k*-means clusters (0.5 values are considered of moderate strength). Despite these results, empirical randomization tests showed that the probability of obtaining these clusters by chance was in both cases 0 and therefore the clusters were statistically significant.

Fig 1 shows the relationship of subject microbial profiles, depicted in rows in the heatmap, with their demographic, medical and clinical characteristics. Subject rows are organized according to hierarchical clustering. Ethnicity, gender, diabetes, CKD and the clinical parameter PD of sampled sites (indicating depth of pockets sampled) do not seem to differ between clusters A and B. On the contrary, those samples with the greatest mean full mouth PD and higher % sites with PD ≥ 5 mm (indicators of disease in the whole mouth) fell into cluster B, suggesting that the clusters observed could be related to the severity and extent of periodontitis. A trend is also evident for subjects in cluster B to have higher % sites with bleeding on probing (BoP), an indicator of whole mouth gingival inflammation. Statistical bivariate correlation analysis between cluster type and each individual demographic, medical and clinical characteristic showed the only significant correlation to be that between cluster type and % sites with PD ≥ 5 mm (Spearman, *r*_*s*_ 0.373, *P* = 0.03). Since most diabetic subjects also suffered from CKD, we also conducted a partial correlation analysis of cluster type and diabetes controlling for CKD, which was also non-significant. A comparison of mean clinical parameters between clusters showed that only full mouth PD and % sites with PD ≥ 5 mm differed in a statistically significant manner (Table 2). This was also true for clusters formed via the k-means method (S1 Table). Although this study was not specifically designed to compare sites as plaque samples were pooled by subject, we observed that with both clustering approaches the clinical parameters specific to the sites sampled per subject (mean PD and CAL of sampled sites) did not differ between clusters (Fig 1 and Table 2).

**Table 2:** Comparison of mean clinical periodontal parameters between subjects in periodontitis clusters A and B (unsupervised hierarchical).

Clinical Characteristic	Cluster A	Cluster B	Statistic
PD (mm)	2.94±0.36	3.48±1.00	*P* = 0.036
CAL (mm)	3.52±0.81	3.88±1.48	NS
BoP (% sites)	38.30±23.02	51.06±26.57	NS
PS (% sites)	66.42±22.88	66.57±23.88	NS
PD ≥ 5 mm (% sites)	11.47±7.52	25.67±23.70	*P* = 0.019
PD sampled sites (mm)	6.53±1.54	6.60±1.24	NS
CAL sampled sites (mm)	7.76±1.85	7.20±1.50	NS

PD: pocket depth; CAL: clinical attachment level; BoP: bleeding on probing; PS: plaque score. Data represent mean ± standard deviation or frequencies (%). Clusters were defined using the unsupervised hierarchical complete linkage method.

### Signature OTUs of the two community types (clusters A and B) and their correlation with periodontitis clinical parameters

**Fig 2:**
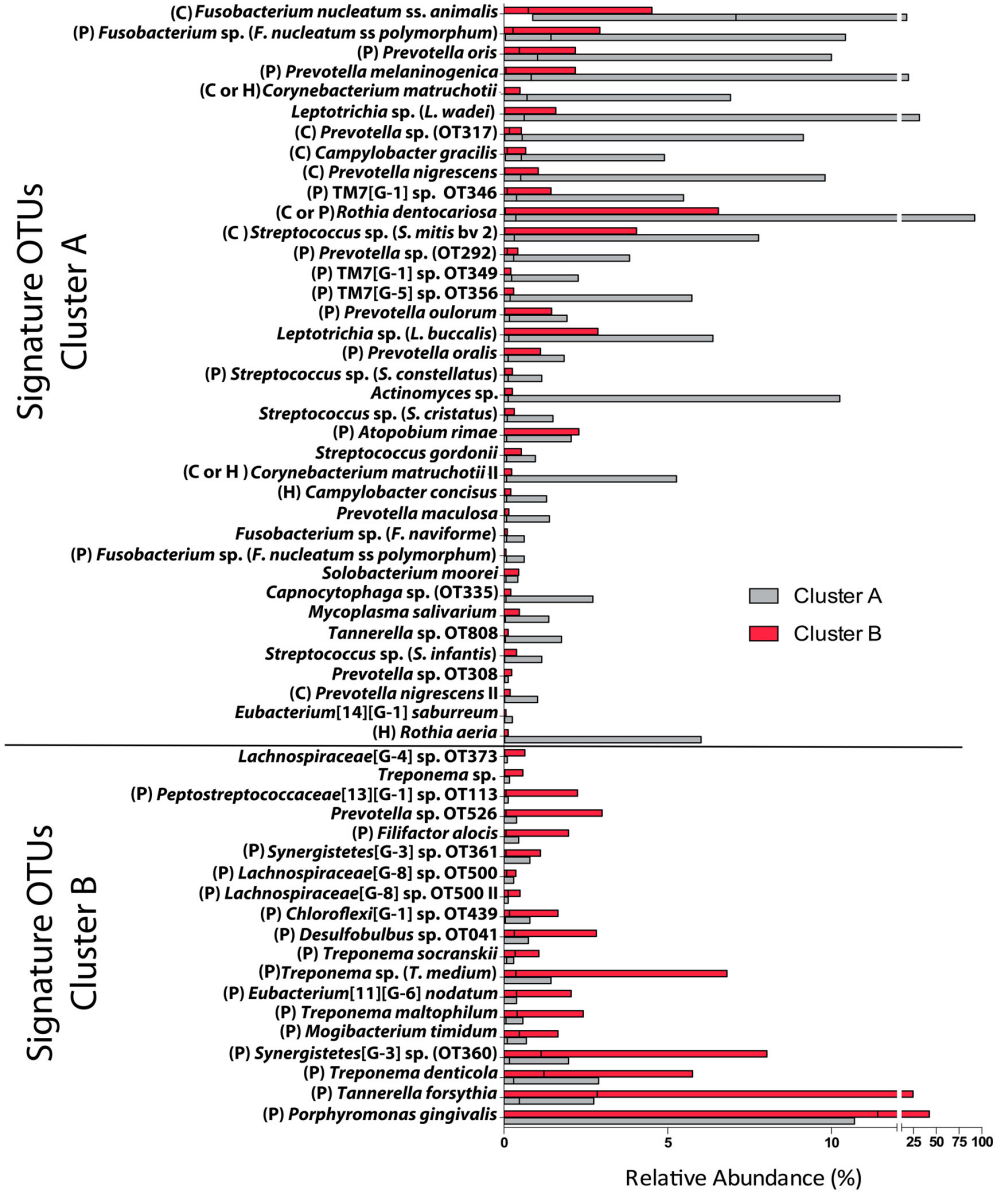
Signature OTUs that define periodontitis clusters A and B (hierarchical). Graph shows OTUs with a statistically significant difference in relative abundance between hierarchical clusters A and B. Bars represent the median and range of relative abundances for each OTU. OTUs increased in Cluster A appear in the top, while OTUs increased in cluster B appear in the bottom. OTUs were labeled according to their association with health or periodontitis considering data from Abusleme et al. [6], Perez-Chaparro et al. [31] and a comparison of our periodontitis samples with HMP healthy subjects (S4 Fig). Taxa were classified as P (periodontitis), H (health) and C (core species), according to at least one study. If a disagreement existed between studies, taxa were labeled under more than one category.

Fig 2 shows OTUs with statistically significant differences in abundance between clusters A and B (hierarchical method). OTUs significantly different between k-means clusters largely agreed with those discriminating hierarchical clusters (S2 Table).

To better interpret these results we annotated signature taxa, depicted in Fig 2, as associated with health or periodontitis or as core species (equally prevalent in health and disease), according to our previous study [6] and a recently published systematic review [31]. We also complemented these published data on the association of taxa with health and periodontitis by performing a comparison of our periodontitis cohort (n = 34) to healthy subjects sequenced by the Human Microbiome Project (HMP) (n = 79), since not all of the published studies used as reference were conducted in the US population (see S1 Supporting Methods). As expected, this analysis showed that communities from health and periodontitis formed distinct data clouds in Principal Coordinate Analysis plots (S3 Fig). We also observed separation in these plots between clusters A and B, and between each cluster and the communities from healthy subjects. The health and periodontitis datasets were then used to define the subgingival microbiomes associated with each condition (S4 Fig), using a methodology similar to that previously employed by our group when characterizing a Chilean cohort [6]. Only species present in a majority of subjects (>50%) in either the healthy or periodontitis groups were taken into account. Species were then categorized based on differences in prevalence and relative abundance between groups. The green shapes in S4 Fig contain species associated with periodontal health, while the red shapes contain species associated with periodontitis. As previously described [6], we also found that some species were equally prevalent in health and periodontitis, designating these as core taxa (yellow, gray and orange shapes). We acknowledge, however, that plaque samples from the HMP and our cohort were processed using different DNA extraction and PCR procedures. Despite possible methodological biases, the health- and disease-associated species identified in this study mostly agreed with those reported previously [6, 7, 31], likely because bias introduced by different processing methodologies was small compared to differences between health and periodontitis.

Interestingly, after annotating the signature OTUs for clusters A and B, depicted in Fig 2, as core, health- or periodontitis-associated, we observed that the signature OTUs for cluster A were a mixture of health-associated, core and disease-associated species, while signature OTUs for cluster B exclusively corresponded to periodontitis-associated species. Among signature OTUs for cluster B were taxa with strong historical associations with periodontitis etiopathology such as *P*. *gingivalis*, *T*. *forsythia* and *T*. *denticola*.

A possible correlation between the combined relative abundance of hierarchical cluster A or cluster B signature OTUs and demographic, medical and clinical characteristics was then evaluated (Table 3). The total abundance of signature OTUs for cluster A negatively correlated with full mouth PD and % of sites with PD ≥ 5 mm, while the opposite correlation was seen for the total abundance of cluster B signature OTUs and % of sites with PD ≥ 5 mm. No correlations between demographic or medical parameters and the total abundance of signature OTUs for clusters A or B were observed.

**Table 3:** Spearman rank order correlation tests between demographic and clinical characteristics of subjects with periodontitis and the total relative abundance of signature OTUs for clusters A and B (hierarchical).

Demographic/Clinical Characteristic	Cluster A Signature OTUs (%)	Cluster B Signature OTUs (%)
Age	0.205 (0.244)	-0.287 (0.100)
Gender	-0.163 (0.357)	0.194 (0.270)
Ethnicity (white, non-white)	0.246 (0.160)	-0.78 (0.661)
Diabetes	0.106 (0.552)	-0.048 (0.787)
CKD	0.159 (0.369)	-0.189 (0.285)
Number of teeth	-0.015 (0.932)	-0.127 (0.473)
PD (mm)	**-0.458 (0.006)**	0.340 (0.049)
CAL (mm)	-0.096 (0.589)	0.104 (0.557)
BoP (% sites)	-0.288 (0.099)	0.388 (0.023)
PS (% sites)	-0.049 (0.782)	0.229 (0.192)
PD ≥ 5 mm (% sites)	**-0.529 (0.001)**	**0.500 (0.003)**
CAL ≥ 5 mm (% sites)	-0.71 (0.689)	0.133 (0.452)
PD sampled sites (mm)	-0.262 (0.135)	0.319 (0.066)
CAL sampled sites (mm)	0.066 (0.710)	0.093 (0.602)

Data correspond to Spearman rank order correlation coefficients (*r*_*s*_) with respective *P* values in parenthesis. Bold caption indicates a statistically significant correlation after multiple comparison adjustment.

Individual correlations between abundances of the top 300 OTUs and full mouth PD, % of sites with PD ≥ 5 mm, BoP and PD of sampled sites were also evaluated. S3–S6 Tables show results from tests with *P* values less than 0.05 and those significant after multiple comparison adjustment. Although some interesting trends were observed, the only correlations significant after multiple test correction were a positive correlation of *Treponema maltophilum* with BoP and *Fusobacterium nucleatum ss*. *vincentii* with % of sites with PD ≥ 5 mm.

## Discussion

The goal of this study was to explore the existence of discrete subgingival microbiome community types in subjects with periodontitis, evaluating whether such communities were related to host and disease characteristics. Results showed subjects tended to separate into two clusters, which were significantly different despite low cohesion and separation metrics, with type B communities correlating with greater periodontitis extent. Although clusters were consistent across methodologies, an explanation for their low cohesion and separation could be the limited number of subjects evaluated (n = 34). It is possible that inclusion of a greater number of subjects would improve clustering metrics, since partitioning confidence has been shown to increase as a function of sample size [1]. Oral communities, however, have also been reported to be more homogeneous across subjects than communities at other body sites, and hence different community types in the oral cavity may not be clearly demarcated [1].

The only previous study, to our knowledge, reporting a cluster analysis of subjects with periodontitis is that of Teles et al. [20], who also found distinct community types across subjects. Although in the mentioned study the technique used to characterize the microbiota included only 40 species, the composition of some of the clusters reported by Teles et al. [20], resembles the two community types found here, with some clusters showing high abundance of red complex species, while *Prevotella* species dominated other clusters, in a pattern similar to communities B and A, respectively, in the current study.

The temporal relationship and clinical significance of the periodontitis clusters reported here needs to be further explored. Subjects in cluster A had communities with higher proportions of health-associated, core and some periodontitis-associated species and a lower number of sites with periodontal breakdown than subjects in cluster B. It is thus possible that cluster A communities are closer to a healthy microbiome than cluster B communities, which were exclusively enriched for periodontitis-associated taxa. S3 Fig explores this hypothesis showing the spatial relationship of the periodontitis clusters found here to the healthy HMP samples also included in our analyses. PCoA based on community structure (θYC index plot, S3 Fig middle panel) shows that indeed cluster A samples are the closest to healthy samples. Cluster A communities could thus represent a temporal transition from health to widespread disease, or alternatively, they constitute the end point of a different community selection process associated with a localized pathological entity. It is also worth noting that not all subjects in cluster B showed widespread periodontitis. However, due to the positive correlation of cluster B signature species with disease extent, it is likely that subjects in cluster B with low number of sites affected are at greater risk for future widespread periodontal breakdown than subjects harboring cluster A communities. There is thus a need to conduct longitudinal evaluations of periodontitis incidence and progression to discern the association of the periodontitis community types reported here with risk for periodontal breakdown. Establishing risk markers not only at a species but also at a community level could represent a useful personalized preventive tool.

A previous study by Zhou et al. [1] explored variability among oral sites in a large number of healthy subjects from the HMP cohort. Interestingly, these researchers also found a tendency for two subgingival community types to exist in health, one of them characterized by higher abundance of the periodontitis-associated genera *Porphyromonas* and *Treponema*. To further explore at a species-level the relationship among the two community types found in health by Zhou *et al*. [1] and those found in periodontitis by our study, we performed unsupervised hierarchical clustering of the 79 HMP subgingival plaque samples included here. This analysis confirmed a tendency for two clusters, a large (L) and a small (S), to form (S5 Fig). Discriminant phylotypes are depicted in S6 Fig, which shows, in agreement with the genus-level analysis of Zhou et al. [1], that periodontitis-associated species of *Treponema* and *Porphyromonas* are increased in the large healthy cluster L. However, this large cluster was also enriched for other periodontitis-associated and core taxa. The small cluster S, on the contrary, was exclusively enriched for health-associated taxa. The relationship of these health clusters with the periodontitis clusters reported here is shown in the right panel of S3 Fig, confirming that, indeed, subjects in the small health cluster are the furthest apart from subjects in periodontitis cluster B. This analysis suggests that these clusters represent a progression of microbiome shifts from health (small cluster HMP) to widespread disease (cluster B perio).

An interesting finding of this study was that the mean PD of sampled sites, which were the two deepest pockets in each subject, did not show a strong relationship to microbial profiles, contrary to subject-level disease indicators, such as the number of sites affected. That is, the depths of pockets sampled could not explain the community types found. We acknowledge, however, that this study was not designed to evaluate if variability in pocket depths within subjects correlated with microbiome profiles, as plaque from the two sampled sites was pooled. Despite this limitation, it was interesting to consider mean clinical parameters of sampled sites in the analyses as subjects displayed great variability in disease presentation, with no correlation between mean PD of sampled sites and % sites affected or subject-level mean PD (ie. the deepest pockets sampled did not originate in all cases from subjects with widespread disease and vice versa (see Fig 1)). The fact that the number of sites affected had a higher correlation with microbial profiles than the depth of the pockets sampled, suggests global (whole mouth) rather than localized microbial dysbiosis. The question of which environmental pressures selected for each community type, however, remains unsolved, but our data suggest a host-level, rather than a site-level effect. For instance, although the signature OTUs for type A communities included species known to be facultative anaerobes, while type B signature OTUs were mostly strict anaerobes, it is unlikely that site-specific oxygen availability (as determined by pocket depth) was the environmental pressure selecting for the two community types since, as mentioned above, the depth of the pockets sampled was strikingly similar between clusters (see Table 2). Therefore, it is more likely that the host phenotype selects for different types of communities (promoting enrichment of different members). Perhaps differences in inflammatory responses and therefore variable volumes and composition of gingival crevicular exudate could create distinct nutritional environments, enriching for specific communities. A second possibility to explain different community types in periodontitis is that inter-species interactions shape community development. For example, the levels of the type A signature OTU *Streptococcus cristatus* have been shown to negatively correlate to the levels of the type B signature OTU *P*. *gingivalis* in subgingival plaque of subjects with periodontitis [32]. This negative correlation appears to be related to bacterial antagonism in which a surface-associated arginine deiminase of *S*. *cristatus* represses production of *P*. *gingivalis* long fimbriae affecting biofilm formation in vitro and oral colonization in a murine model [32, 33]. Further studies, however, are needed to confirm if indeed different community types do not exist at different sites within the same individual and to evaluate the importance of habitat-filtering (the environment) versus inter-species interactions as selective pressures for selection of community types. Moreover, it is not clear if the different community types observed are stable end points or stages within a continually evolving dysbiotic process.

The relationship of clinical phenotypes and biological markers related to periodontitis etiopathology was previously explored by Offenbacher *et al*. [17], who divided periodontitis subjects according to their BoP scores, a marker for gingival inflammation. Subjects with >50% of sites with BoP showed increased *P*. *gingivalis* load in plaque and higher serum IgG levels when compared to subjects with less BoP. In the present study, BoP only showed a trend to correlate with the microbiome (results were not statistically significant). In contrast, the % of sites with PD ≥ 5 mm is a parameter that should be explored as a clinical marker of biologically distinct periodontitis processes as it showed a stronger relationship to the two community types found. Moreover, treatment outcomes of subjects harboring each community type warrant further evaluation.

Our top-down data analyses suggested that diabetes and CKD, two systemic conditions that affect periodontitis prevalence are not related to the two community types uncovered in periodontitis (Fig 1). To further explore if diabetes and CKD determine similarity among subgingival microbiome communities, we also used β-diversity metrics to measure distance among communities of individuals positive and negative for these conditions (see S1 Supporting Methods and S7 Fig). This analysis also suggested that diabetes and CKD status do not correlate with periodontitis microbiome profiles. One limitation of this study, however, is that most diabetic subjects included also suffered from CKD. Although a partial correlation analysis of community types and diabetes, controlling for CKD, suggested no correlation between diabetes and clusters A and B, a clear conclusion on whether diabetes affects subgingival communities in periodontitis necessitates a targeted study with a cohort of diabetic patients suffering from no other potentially confounding condition. In this respect, previous work by Zhou et al. [34] has shown that diabetes indeed influences subgingival microbiome profiles. The discrepancy between Zhou et al. [34] and our findings needs further exploration, as these authors did not control for disease severity and extent in their study, while we found that disease severity/extent rather than medical characteristics influence the subgingival microbiome.

One caveat of the methodology in the current study is the relatively liberal inclusion criterion regarding antibiotic use. Systemic antibiotic intake is likely to modify the subgingival microbiome, with evidence from gut communities suggesting that full recovery does not occur even after the usual 6-month antibiotic-free period commonly used by most studies [21, 22]. Antibiotic use, however, is not a likely determinant of the community types found as subjects with CKD, who experience the most frequent antibiotic intake, did not fall into a specific cluster.

A great amount of evidence from studies prior to the high throughput sequencing era linked the red complex species *P*. *gingivalis*, *T*. *forsythia* and *T*. *denticola* to periodontitis [15, 16]. All studies using 16S rRNA gene sequencing, including this one, confirm that indeed these three species become dominant members in some communities in periodontitis. However, we observed differences in the abundance of these species, when compared to Chilean subjects previously studied by our group [6]. In the present study, *P*. *gingivalis* and *T*. *forsythia* appeared among the most abundant periodontitis-associated phylotypes (S4 Fig). For instance, although only about two thirds of subjects with periodontitis harbored *P*. *gingivalis*, this species ranked among the top three most abundant in almost all subjects in which it was present, reaching 42% of reads in one individual. In contrast, we had seen that periodontitis communities of Chilean subjects were dominated by spirochaetes, while *P*. *gingivalis* and *T*. *forsythia* were present at low abundance [6]. Moreover, in contrast to the very low prevalence of *Prevotella* in the Chilean cohort, the present study found a great variety of *Prevotella* species at high prevalence and in some cases in high abundance (S4 Fig). These results are in agreement with those from Griffen et al. [7] and confirm the importance of certain *Prevotella* spp. in periodontitis, at least in US subjects. A greater abundance of *Prevotella* spp. in US versus Chilean subjects was also observed by Haffajee et al. [35]. In summary, although geographical location can influence species prevalence and their representation in communities, a consistent picture is emerging on which are the species associated with periodontitis across populations. Community types, however, should be considered when evaluating species abundances in different periodontitis cohorts.

In conclusion, this study found two types of microbial communities exist in periodontitis. These community types correlated with clinical indicators of disease extent suggesting that widespread and localized forms of periodontitis are associated with different microbiomes. No demographic or medical condition evaluated here showed a significant association with community types. We nevertheless acknowledge that our “top down” study design provides only preliminary evidence due to its exploratory nature. Among all the variables evaluated, our analysis prioritizes, however, the need to conduct large, targeted and well-controlled studies, specifically designed to clarify the association among subgingival community types, disease extent indicators, future disease risk and therapy outcomes.

## Supporting Information

S1 Fig Dendograms depicting unsupervised hierarchichal clustering (complete linkage) of subjects according to OTU relative abundance profiles. (PDF)

S2 Fig Two-dimensional scatter plot showing clustering of samples according to k-means. (PDF)

S3 Fig Comparison of periodontitis communities from this study (n = 34) with HMP healthy subjects (n = 79). (PDF)

S4 Fig Association of subgingival microbiome species with health and periodontitis. (PDF)

S5 Fig Unsupervised hierarchical clustering of subgingival microbiome samples from 79 healthy HMP subjects. (PDF)

S6 Fig Phylotypes enriched in HMP clusters. (PDF)

S7 Fig CKD and diabetes do not drive clustering of periodontitis microbial communities. (PDF)

S1 Table Comparison of mean clinical periodontal parameters between subjects in periodontitis clusters A and B (k-means). (DOCX)

S2 Table Comparison of signature OTUs (with increased relative abundance) in hierarchical and k-means clusters A and B. (DOCX)

S3 Table Spearman Rank Order correlation tests between relative abundances of individual OTUs (top 300 most abundant) and mean full mouth PDs. (DOCX)

S4 Table Spearman Rank Order correlation tests between relative abundances of individual OTUs (top 300 most abundant) and % sites with BoP. (DOCX)

S5 Table Spearman Rank Order correlation tests between relative abundances of individual OTUs (top 300 most abundant) and % of sites with PD ≥ 5mm. (DOCX)

S6 Table Spearman Rank Order correlation tests between relative abundances of individual OTUs (top 300 most abundant) and PDs of sampled sites. (DOCX)

S1 Supporting Methods (PDF)
