# Correction: Microbiome Profiles in Periodontitis in Relation to Host and Disease Characteristics

**DOI:** 10.1371/journal.pone.0148893

**Published:** 2016-02-03

**Authors:** Bo-Young Hong, Michel V. Furtado Araujo, Linda D. Strausbaugh, Evimaria Terzi, Effie Ioannidou, Patricia I. Diaz

The fourth paragraph of the Discussion section incorrectly characterizes large cluster L and small cluster S. It should read:

Discriminant phylotypes are depicted in S6 Fig, which shows, in agreement with the genus-level analysis of Zhou et al. [1], that periodontitis-associated species of *Treponema* and *Porphyromonas* are increased in the small healthy cluster S. However, this small cluster was also enriched for other periodontitis-associated and core taxa. The large cluster L, on the contrary, was exclusively enriched for health-associated taxa. The relationship of these health clusters with the periodontitis clusters reported here is shown in the right panel of S3 Fig, confirming that, indeed, subjects in the large health cluster are the furthest apart from subjects in the periodontitis clusters when PC1 is considered. This analysis suggests that these clusters represent a progression of microbiome shifts from health (large cluster HMP) to widespread disease (cluster B perio).
