# Supplementary material for: Microbiome Profiles in Periodontitis in Relation to Host and Disease Characteristics
Source: PLoS One. 2015 May 18;10(5):e0127077. doi: 10.1371/journal.pone.0127077 (PMC4436126; doi:10.1371/journal.pone.0127077)
Supplement: S1 Fig — (PDF) [file pone.0127077.s001.pdf]

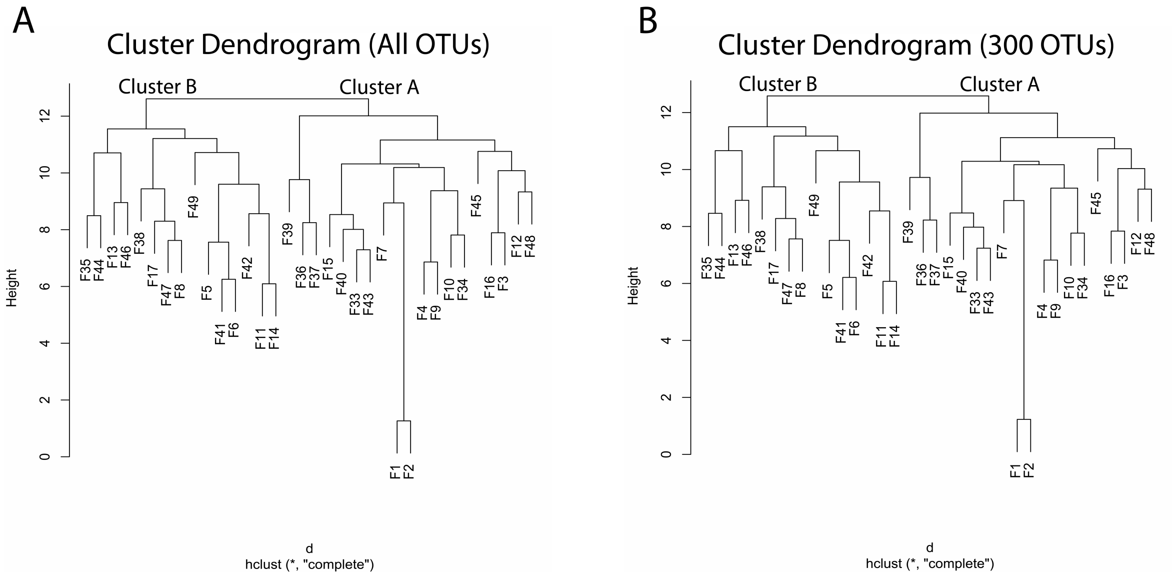

Figure S1. Dendograms depicting unsupervised hierarchichal clustering (complete linkage) of subjects according to OTU relative abundance profiles. Clustering based on all OTUs is shown in A and clustering based on the 300 most abundant OTUs is shown in B. Identical results indicate the top 300 OTUs drive clustering of samples.
