# Supplementary material for: Microbiome Profiles in Periodontitis in Relation to Host and Disease Characteristics
Source: PLoS One. 2015 May 18;10(5):e0127077. doi: 10.1371/journal.pone.0127077 (PMC4436126; doi:10.1371/journal.pone.0127077)
Supplement: S2 Fig — (PDF) [file pone.0127077.s002.pdf]

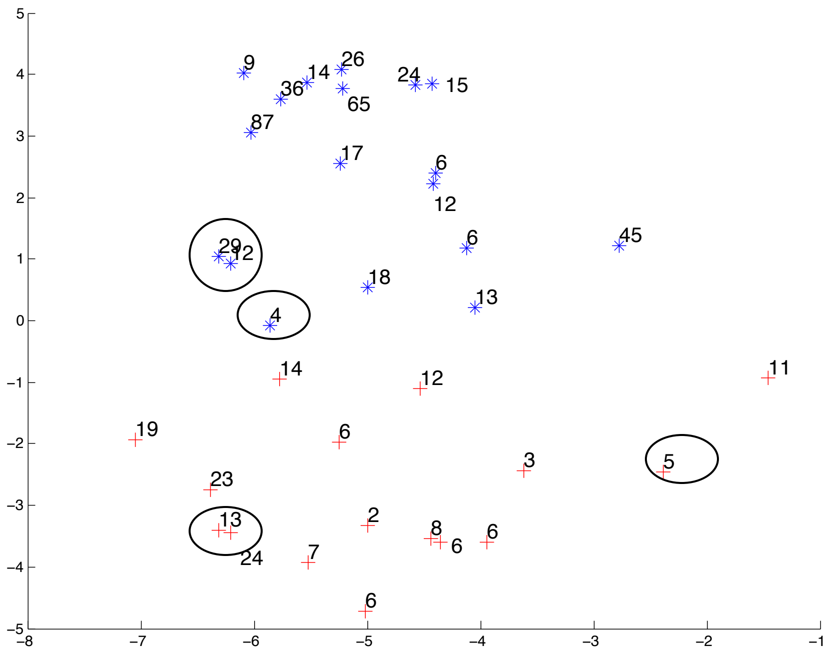

Figure S2. Two-dimensional scatter plot showing clustering of samples according to k-means. Two dimensional scatter plot was created after Singular Value Decomposition (SVD) of the matrix containing the OTU relative abundance data. X-axis represents component 1 and y-axis represents component 2. Data points are colored according to placement of subjects in k-means clusters with cluster A in red and cluster B in blue. Data points encircled were those that fell into a different cluster when using the unsupervised hierarchical method. Numerical values besides each data point represent % sites with PD $\geq$ 5 mm.
