# Supplementary material for: Microbiome Profiles in Periodontitis in Relation to Host and Disease Characteristics
Source: PLoS One. 2015 May 18;10(5):e0127077. doi: 10.1371/journal.pone.0127077 (PMC4436126; doi:10.1371/journal.pone.0127077)
Supplement: S3 Fig — (PDF) [file pone.0127077.s003.pdf]

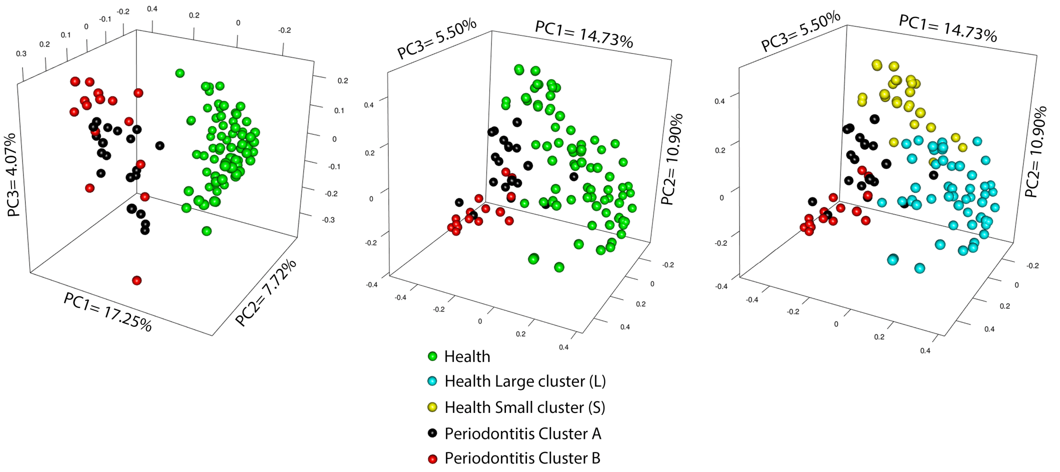

Figure S3. Comparison of periodontitis communities from this study (n=34) with HMP healthy subjects (n=79). Graphs are principal coordinate analysis plots. The plot on the left was based on the Jaccard Index, which measures distance among samples according to taxa prevalence. The plot in the middle was based on the θ_{YC} index, which measures distance among samples according to community structure. Using both metrics, healthy subjects (green), periodontitis cluster A (black) and periodontitis cluster B (red) subjects clustered separately (AMOVA $P<0.001$ between periodontitis and health, $P<0.001$ between periodontitis cluster A and health, $P<0.001$ between periodontitis cluster B and health, and $P=0.004$ between periodontitis cluster A and B for Jaccard-based data clouds and $P<0.001$ for θ_{YC}-based data clouds). The right PCoA plot shows health HMP samples colored according to clusters reported in S5 Fig.
