# Supplementary material for: Microbiome Profiles in Periodontitis in Relation to Host and Disease Characteristics
Source: PLoS One. 2015 May 18;10(5):e0127077. doi: 10.1371/journal.pone.0127077 (PMC4436126; doi:10.1371/journal.pone.0127077)
Supplement: S4 Fig — (PDF) [file pone.0127077.s004.pdf]

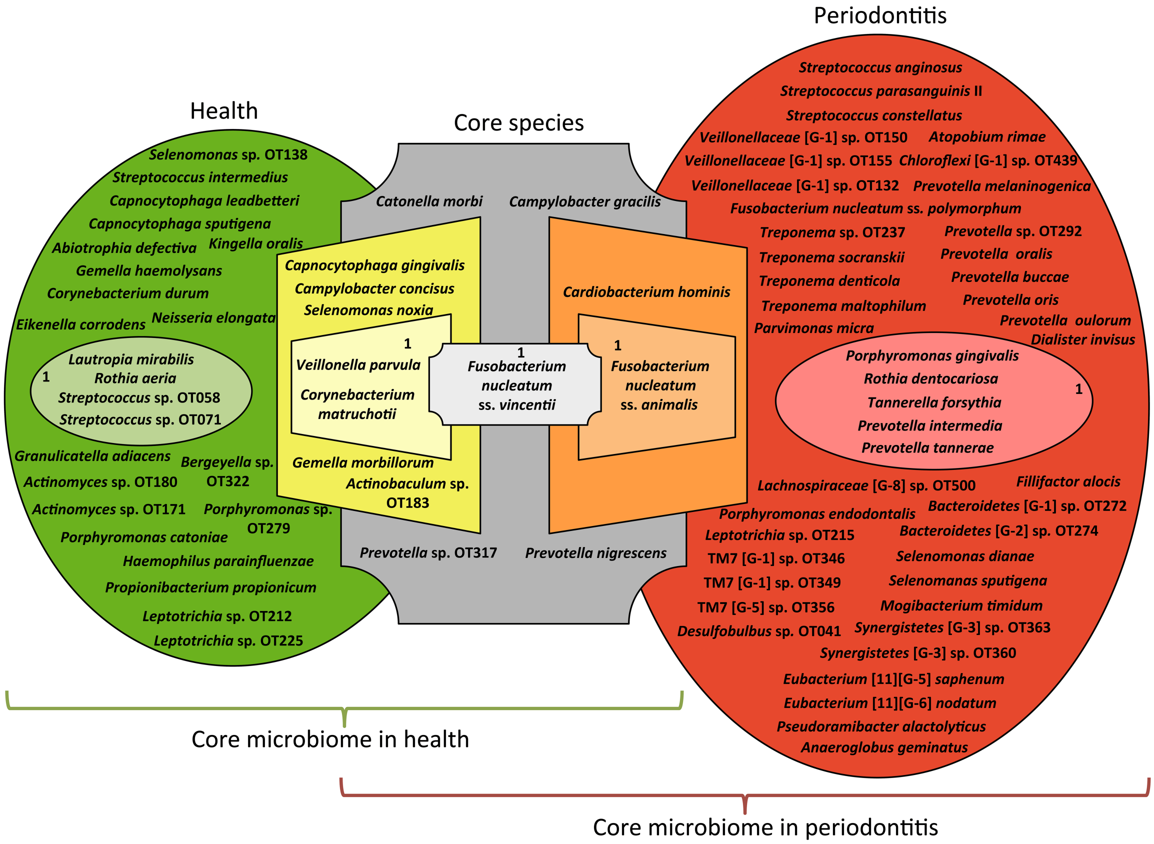

Figure S4. Association of subgingival microbiome species with health and periodontitis. Graph shows species present in at least 50% of subjects in health or periodontitis. Species in the gray shape were found at equal prevalence and relative abundance in health and periodontitis. Species in the yellow shape were equally prevalent in health and periodontitis but had higher relative abundance in health. Species in the orange shape were equally prevalent in health and periodontitis but had higher relative abundance in periodontitis. The gray, yellow and orange shapes represent the “core species”. Species in the green shape had greater prevalence and relative abundance in health are therefore “health-associated”. Species in the red shape were found at greater prevalence and relative abundance in periodontitis are therefore “periodontitis-associated”. Species placed in the inner shapes labeled with 1 were those found at >2% mean relative abundance, indicating the most numerous species from each category.
