# Supplementary material for: Microbiome Profiles in Periodontitis in Relation to Host and Disease Characteristics
Source: PLoS One. 2015 May 18;10(5):e0127077. doi: 10.1371/journal.pone.0127077 (PMC4436126; doi:10.1371/journal.pone.0127077)
Supplement: S5 Fig — (PDF) [file pone.0127077.s005.pdf]

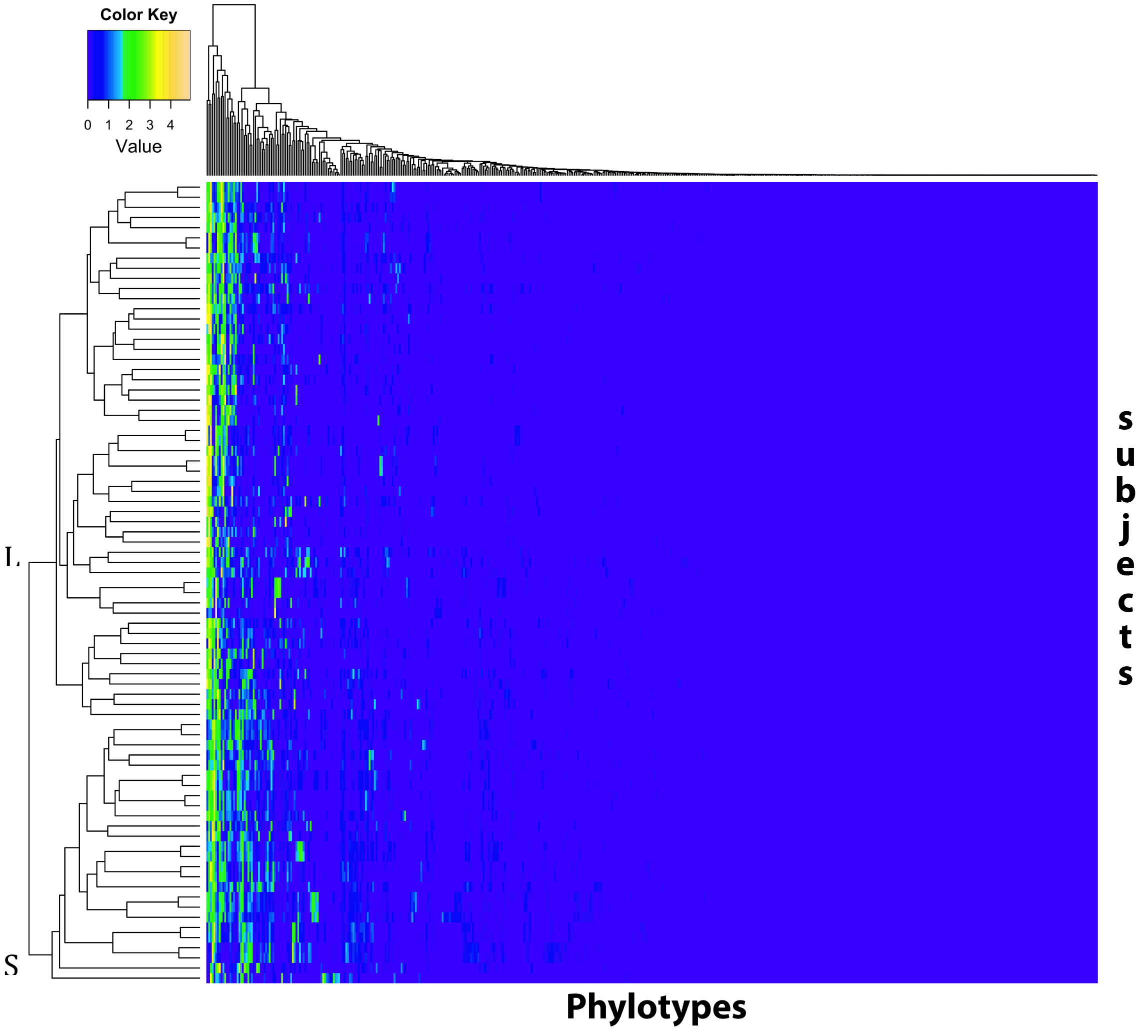

Figure S5. Unsupervised hierarchical clustering of subgingival microbiome samples from 79 healthy HMP subjects. Graph shows results of unsupervised hierarchical clustering (complete linkage) of the transformed relative abundances (inverse sine method) of all phylotypes found. Subjects (in rows) tended to form two distinct clusters, Large (L) and Small (S).
