# Supplementary material for: Microbiome Profiles in Periodontitis in Relation to Host and Disease Characteristics
Source: PLoS One. 2015 May 18;10(5):e0127077. doi: 10.1371/journal.pone.0127077 (PMC4436126; doi:10.1371/journal.pone.0127077)
Supplement: S6 Fig — (PDF) [file pone.0127077.s006.pdf]

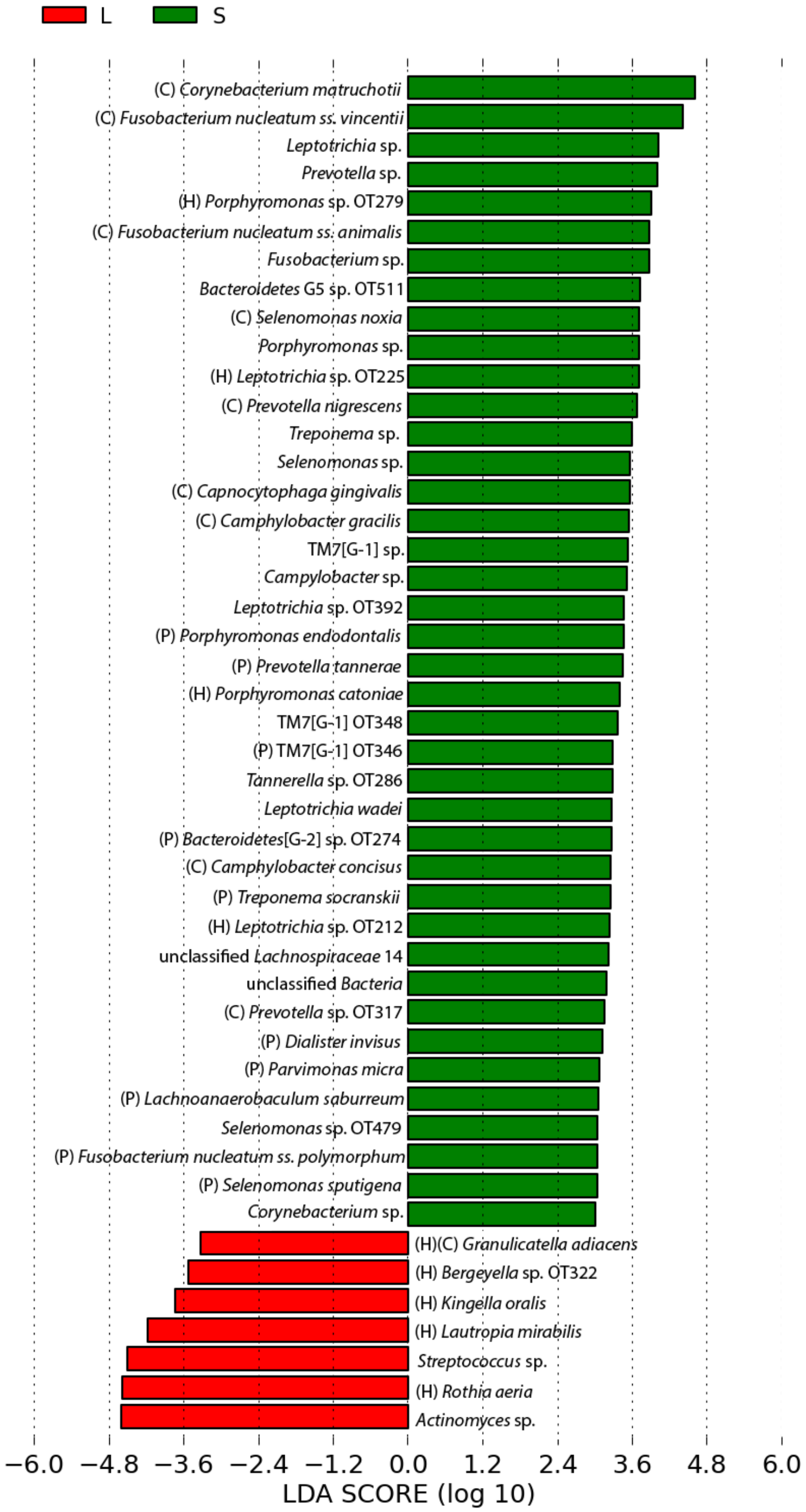
L
S
(C) Corynebacterium matruchotii
(C) Fusobacterium nucleatum ss. vincentii
Leptotrichia sp.
Prevotella sp.
(H) Porphyromonas sp. OT279
(C) Fusobacterium nucleatum ss. animalis
Fusobacterium sp.
Bacteroidetes G5 sp. OT511
(C) Selenomonas noxia
Porphyromonas sp.
(H) Leptotrichia sp. OT225
(C) Prevotella nigrescens
Treponema sp.
Selenomonas sp.
(C) Capnocytophaga gingivalis
(C) Camphylobacter gracilis
TM7[G-1] sp.
Campylobacter sp.
Leptotrichia sp. OT392
(P) Porphyromonas endodontalis
(P) Prevotella tannerae
(H) Porphyromonas catoniae
TM7[G-1] OT348
(P) TM7[G-1] OT346
Tannerella sp. OT286
Leptotrichia wadei
(P) Bacteroidetes[G-2] sp. OT274
(C) Camphylobacter concisus
(P) Treponema socranskii
(H) Leptotrichia sp. OT212
unclassified Lachnospiraceae 14
unclassified Bacteria
(C) Prevotella sp. OT317
(P) Dialister invisus
(P) Parvimonas micra
(P) Lachnoanaerobaculum saburreum
Selenomonas sp. OT479
(P) Fusobacterium nucleatum ss. polymorphum
(P) Selenomonas sputigena
Corynebacterium sp.
(H)(C) Granulicatella adiacens
(H) Bergeyella sp. OT322
(H) Kingella oralis
(H) Lautropia mirabilis
Streptococcus sp.
(H) Rothia aeria
Actinomyces sp.
−6.0
−4.8
−3.6
−2.4
−1.2
0.0
1.2
2.4
3.6
4.8
6.0
LDA SCORE (log 10)

S6 Figure. Phylotypes enriched in HMP clusters. Graph shows phylotypes with a statistically significant difference in relative abundance between the large and small HMP clusters depicted in S5 Fig. Statistical significance was determined using LEfSe with alpha values set at 0.01 and the threshold for the logarithmic linear discriminant analysis (LDA) score for discriminative features set at 3.0. Phylotypes were labeled according to their association with health or periodontitis considering data from Abusleme et al. [6], Perez-Chaparro et al. [31] and a comparison of our periodontitis samples with HMP healthy subjects (S4 Fig). Taxa were classified as P (periodontitis), H (health) and C (core species), according to at least one study. If a disagreement existed between studies, taxa were labeled under more than one category.
