# Supplementary material for: Microbiome Profiles in Periodontitis in Relation to Host and Disease Characteristics
Source: PLoS One. 2015 May 18;10(5):e0127077. doi: 10.1371/journal.pone.0127077 (PMC4436126; doi:10.1371/journal.pone.0127077)
Supplement: S7 Fig — (PDF) [file pone.0127077.s007.pdf]

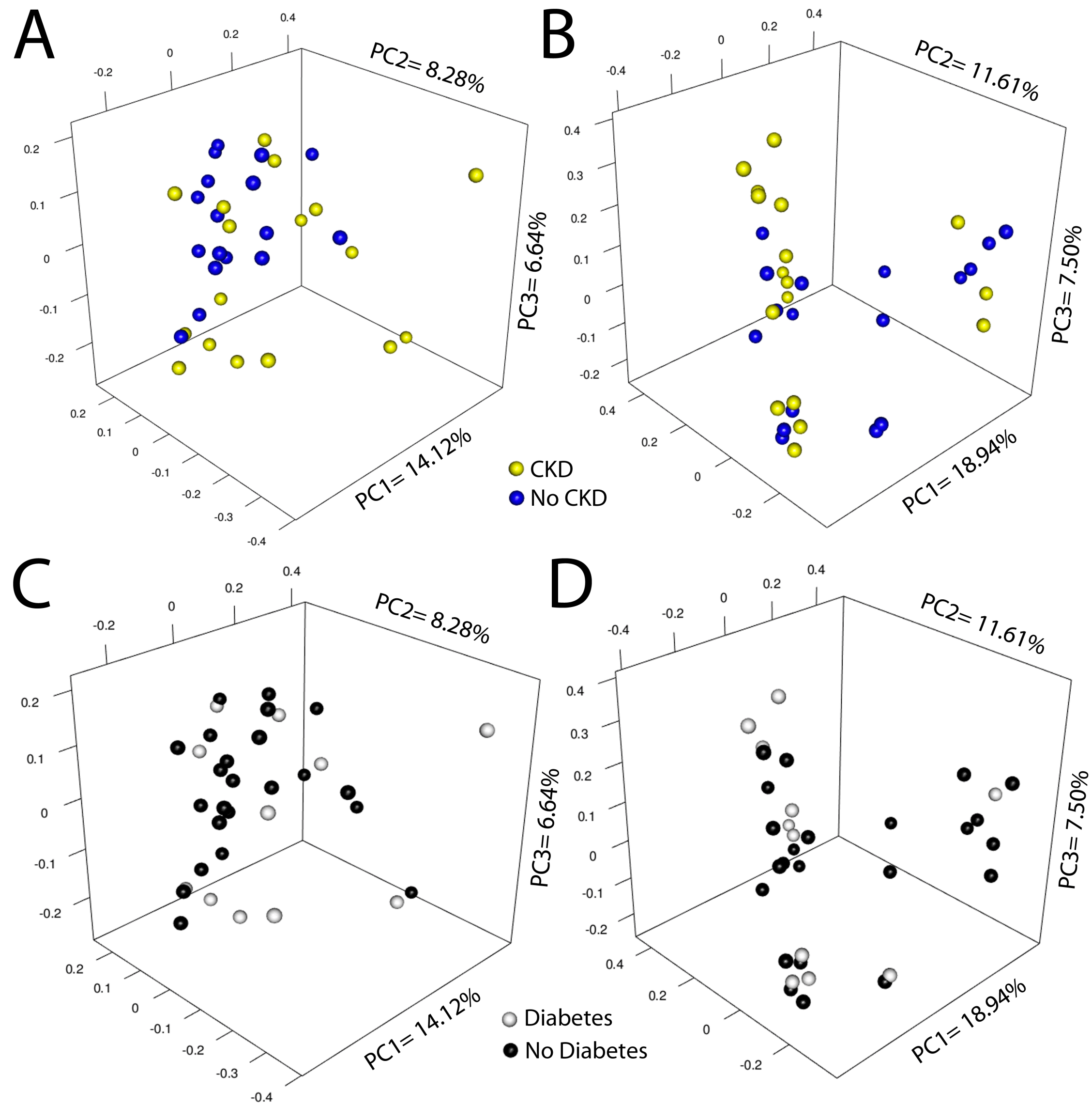

Figure S7. CKD and diabetes do not drive clustering of periodontitis microbial communities. Graphs represent PCoA plots of distances among samples. Graphs A and C are based on the Jaccard index and therefore indicate differences in community composition. Graphs B and D are based on the θYC index and therefore indicate differences in community structure. Amova comparisons were not statistically significant (A, Jaccard CKD vs No CKD: P= 0.89; B, θYC CKD vs No CKD: P= 0.436; C, Jaccard Diabetes vs No Diabetes: P= 0.463; D, θYC Diabetes vs No Diabetes: P= 0.334).
