# Supplementary material for: Microbiome Profiles in Periodontitis in Relation to Host and Disease Characteristics
Source: PLoS One. 2015 May 18;10(5):e0127077. doi: 10.1371/journal.pone.0127077 (PMC4436126; doi:10.1371/journal.pone.0127077)
Supplement: S1 Table — (DOCX) [file pone.0127077.s008.docx]

**S1 Table.** Comparison of mean clinical periodontal parameters between subjects in periodontitis clusters A and B (k-means).

| Clinical Characteristic | Cluster A | Cluster B | Statistic |
| --- | --- | --- | --- |
| Full mouth PD | 2.88±0.32 | 3.45±0.91 | *P*=0.024 |
| Full mouth CAL | 3.52±0.86 | 3.82±1.37 | NS |
| BoP (% sites) | 38.27±24.23 | 48.96±25.45 | NS |
| PS (% sites positive) | 68.57±23.02 | 64.63±23.42 | NS |
| Sites ≥ PD 5 mm (%) | 10.27±6.65 | 24.37±22.00 | *P*=0.019 |
| PD sampled sites | 6.28±1.61 | 6.81±1.18 | NS |
| CAL sampled sites | 7.63±2.00 | 7.42±1.44 | NS |

PD: pocket depth; CAL: clinical attachment level; BoP: bleeding on probing; PS: plaque score. Data represent mean ± standard deviation or frequencies (%). Clustering was performed using a k-means method assuming 2 clusters within the data.
