# Supplementary material for: Microbiome Profiles in Periodontitis in Relation to Host and Disease Characteristics
Source: PLoS One. 2015 May 18;10(5):e0127077. doi: 10.1371/journal.pone.0127077 (PMC4436126; doi:10.1371/journal.pone.0127077)
Supplement: S2 Table — (DOCX) [file pone.0127077.s009.docx]

**S2 Table.** Comparison of signature OTUs (with increased relative abundance) in hierarchical and k-means clusters A and B.

| **OTU name** | **Increased in hierarchical**  **cluster B** | **Increased in k-means**  **cluster B** | **Increased in hierarchical**  **cluster A** | **Increased in k-means**  **cluster A** |
| --- | --- | --- | --- | --- |
| *Porphyromonas gingivalis* | yes | yes |  |  |
| *Tannerella forsythia* | yes | yes |  |  |
| *Treponema denticola* | yes | yes |  |  |
| *Synergistetes*[G-3] sp*.* (*Fretibacterium sp.* OT 360) | yes | yes |  |  |
| *Treponema* sp. (*Treponema medium*) | yes | yes |  |  |
| *Desulfobulbus* sp. OT 041 | yes | yes |  |  |
| *Treponema maltophilum* | yes | yes |  |  |
| *Mogibacterium timidum* | yes | yes |  |  |
| *Eubacterium* [11][G-6] *nodatum* | yes | yes |  |  |
| *Prevotella* sp*.* OT 526 | yes | yes |  |  |
| *Filifactor alocis* | yes | yes |  |  |
| *Choroflexi* [G-1] sp. OT 439 | yes | yes |  |  |
| *Treponema socranskii* | yes | yes |  |  |
| *Peptostreptococcaceae*[13][G-1] sp. OT 113 | yes | yes |  |  |
| *Synergistetes*[G-3] sp. OT 361 | yes | yes |  |  |
| *Lachnospiraceae*[G-8] sp. OT 500 | yes | yes |  |  |
| *Lachnospiraceae*[G-8] sp. OT 500 II | yes | yes |  |  |
| *Lachnospiraceae*[G-4] sp. OT 373 | yes | yes |  |  |
| *Treponema* sp. | yes | yes |  |  |
| *Prevotella intermedia* |  | yes |  |  |
| *Synergistetes*[G-3] sp. OT 363 |  | yes |  |  |
| *Treponema* sp. OT 237 |  | yes |  |  |
| *Porphyromonas endodontalis* |  | yes |  |  |
| *Synergistetes*[G-3] sp. OT 362 |  | yes |  |  |
| *Treponema maltophilum* |  | yes |  |  |
| *Bacteroidetes*[G-3] sp. OT 280 |  | yes |  |  |
| *Treponema parvum* |  | yes |  |  |
| *Veillonellaceae*[G-1] sp. (OT 148) |  | yes |  |  |
| *Fusobacterium* sp. (*F. nucleatum* ss *vincentii*) |  | yes |  |  |
| *Eubacterium*[11][G-6] *minutum* |  | yes |  |  |
| *Clostridiales*[F-1][G-1] sp. OT 093 |  | yes |  |  |
| *Atopobium parvulum* |  | yes |  |  |
| *Fusobacterium nucleatum* ss *animalis* |  |  | yes | yes |
| *Rothia dentocariosa* |  |  | yes | yes |
| *Leptotrichia* sp. (*Leptotrichia wadei*) |  |  | yes | yes |
| *Fusobacterium* sp. (*F. nucleatum* ss *polymorphum*) |  |  | yes | yes |
| *Prevotella melaninogenica* |  |  | yes | yes |
| *Prevotella nigrescens* |  |  | yes | yes |
| *Campylobacter gracilis* |  |  | yes | yes |
| *Prevotella* sp. (OT 292) |  |  | yes | yes |
| *Prevotella oulorum* |  |  | yes | yes |
| *Atopobium rimae* |  |  | yes | yes |
| *Streptococcus* sp. (*S. cristatus*) |  |  | yes | yes |
| *Capnocytophaga* sp. (OT 335) |  |  | yes | yes |
| *Prevotella maculosa* |  |  | yes | yes |
| *Mycoplasma salivarium* |  |  | yes | yes |
| *Gemella morbillorum* |  |  | yes | yes |
| *Fusobacterium* sp. (*F. naviforme*) |  |  | yes | yes |
| *Solobacterium morei* |  |  | yes | yes |
| *Fusobacterium* sp. (*F. nucleatum* ss *polymorphum*) II |  |  | yes | yes |
| *Prevotella nigrescens* II |  |  | yes | yes |
| *Eubacterium*[14][G-1] *saburreum* |  |  | yes | yes |
| *Prevotella* sp. OT 308 |  |  | yes | yes |
| *Prevotella oris* |  |  | yes |  |
| *Prevotella* sp. (OT 317) |  |  | yes |  |
| *Corynebacterium matruchotti* |  |  | yes |  |
| *Streptococcus* sp. (*S. mitis* bv 2 OT) |  |  | yes |  |
| TM7[G-1] sp. OT 346 |  |  | yes |  |
| TM7[G-5] sp. OT 356 |  |  | yes |  |
| *Leptotrichia* sp. (*Leptotrichia buccalis*) |  |  | yes |  |
| *Actinomyce*s sp. |  |  | yes |  |
| *Corynebacterium matruchotti* II |  |  | yes |  |
| TM7[G-1] sp. OT 349 |  |  | yes |  |
| *Prevotella oralis* |  |  | yes |  |
| *Rothia aeria* |  |  | yes |  |
| *Streptococcus* sp. (*S. constellatus* OT 576) |  |  | yes |  |
| *Tannerella* sp. OT 808 |  |  | yes |  |
| *Streptococcus gordonii* |  |  | yes |  |
| *Streptococcus* sp. (*S. infantis* OT 638) |  |  | yes |  |
| *Actinomyces* sp. OT 169 |  |  |  | yes |
| *Megasphaera micronuciformis* |  |  |  | yes |
| *Lachnospiraceae*[G-4] sp. (*Moryella* sp. OT 419) |  |  |  | yes |
| *Campylobacter concisus* |  |  |  | yes |
| *Capnocytophaga granulosa* |  |  |  | yes |
| *Mogibacterium* sp. (*M. diversum* OT 593) |  |  |  | yes |
| *Granulicatella adiacens* |  |  |  | yes |
| *Prevotella salivae* |  |  |  | yes |
| *Actinomyces* sp. (*A. odontolyticus* OT 701) |  |  |  | yes |
| *Clostridiales*[F-2][G-2] sp. OT 085 |  |  |  | yes |
