# Supplementary material for: Microbiome Profiles in Periodontitis in Relation to Host and Disease Characteristics
Source: PLoS One. 2015 May 18;10(5):e0127077. doi: 10.1371/journal.pone.0127077 (PMC4436126; doi:10.1371/journal.pone.0127077)
Supplement: S3 Table — (DOCX) [file pone.0127077.s010.docx]

**S3 Table**. Spearman Rank Order correlation tests between relative abundances of individual OTUs (top 300 most abundant) and mean full mouth PDs. Only OTUs with *P* values < 0.05 are shown. No correlation was significant after multiple test adjustment.

| OTU | Correlation coefficient (*r_s_*) | P-value | Q-value |
| --- | --- | --- | --- |
| *Fusobacterium nucleatum* ss. *vincentii* | 0.540 | 0.001 | 0.0002 |
| *Treponema* sp. | 0.502 | 0.002 | 0.0003 |
| *Eubacterium*[11][G-3] *brachy* | 0.399 | 0.019 | 0.001 |
| *Prevotella* sp. OT473 | 0.385 | 0.025 | 0.002 |
| *Fusobacterium nucleatum* ss. *vincentii* | 0.370 | 0.031 | 0.003 |
| *Aggregatibacter* sp. (*Aggregatibacter aphrophilus*) | 0.369 | 0.032 | 0.003 |
| *Treponema* sp. | 0.358 | 0.037 | 0.004 |
| *Dialister pneumosintes* | 0.342 | 0.048 | 0.005 |
| *Lachnospiraceae*[G-4] sp. (*Moryella* sp. OT419) | -0.448 | 0.008 | 0.001 |
| *Solobacterium moorei* | -0.433 | 0.011 | 0.001 |
| *Prevotella oulorum* | -0.429 | 0.011 | 0.001 |
| *Veillonella parvula* | -0.415 | 0.015 | 0.001 |
| *Streptococcus* sp. (*Streptococcus vestibularis*) | -0.412 | 0.015 | 0.001 |
| *Atopobium parvulum* | -0.400 | 0.019 | 0.002 |
| *Dialister invisus* | -0.396 | 0.021 | 0.002 |
| *Prevotella buccae* | -0.393 | 0.022 | 0.002 |
| *Actinomyces* sp. OT169 | -0.386 | 0.024 | 0.002 |
| *Shuttleworthia satelles* | -0.381 | 0.026 | 0.002 |
| *Prevotella enoeca* | -0.374 | 0.030 | 0.003 |
| *Fusobacterium nucleatum* ss. *animalis* | -0.373 | 0.030 | 0.003 |
| *Streptococcus gordonii* | -0.367 | 0.033 | 0.003 |
| *Propionibacterium* sp. OT194 | -0.365 | 0.034 | 0.003 |
| *Gemella sanguinis* | -0.356 | 0.039 | 0.004 |
| *Prevotella melaninogenica* | -0.352 | 0.041 | 0.004 |
| Bacteroidetes[G-1] sp. OT272 | -0.349 | 0.043 | 0.004 |
| *Leptotrichia* sp. (*Leptotrichia wadei*) | -0.348 | 0.044 | 0.004 |
| *Atopobium rimae* | -0.346 | 0.045 | 0.004 |
| *Prevotella denticola* | -0.342 | 0.048 | 0.005 |
