# Supplementary material for: Microbiome Profiles in Periodontitis in Relation to Host and Disease Characteristics
Source: PLoS One. 2015 May 18;10(5):e0127077. doi: 10.1371/journal.pone.0127077 (PMC4436126; doi:10.1371/journal.pone.0127077)
Supplement: S4 Table — (DOCX) [file pone.0127077.s011.docx]

**S4 Table**. Spearman Rank Order correlation tests between relative abundances of individual OTUs (top 300 most abundant) and % sites with BoP. Only OTUs with *P* values < 0.05 are shown. Bold indicates significant correlations after multiple test adjustment.

| OTU | Correlation coefficient (*r_s_*) | P-value | Q-value |
| --- | --- | --- | --- |
| ***Treponema maltophilum*** | **0.569** | **<0.0001** | **0.0002** |
| *Treponema socranskii* | 0.521 | 0.002 | 0.0003 |
| Bacteroidetes[G-5] sp. OT511 | 0.484 | 0.004 | 0.001 |
| *Prevotella* sp. OT473 | 0.456 | 0.007 | 0.001 |
| *Prevotella* sp. OT304 | 0.441 | 0.009 | 0.002 |
| Synergistetes[G-3] sp. OT361 | 0.439 | 0.009 | 0.001 |
| *Filifactor alocis* | 0.433 | 0.011 | 0.002 |
| *Treponema denticola* | 0.404 | 0.018 | 0.002 |
| *Treponema maltophilum* II | 0.400 | 0.019 | 0.003 |
| *Prevotella maculosa* | 0.392 | 0.022 | 0.003 |
| *Treponema* sp. (*Treponema medium*) | 0.382 | 0.026 | 0.003 |
| *Capnocytophaga* sp. (OT324) | 0.379 | 0.027 | 0.003 |
| *Porphyromonas* sp. (OT275) | 0.375 | 0.029 | 0.004 |
| *Eubacterium*[11][G-6] *nodatum* | 0.373 | 0.030 | 0.004 |
| *Dialister pneumosintes* | 0.367 | 0.033 | 0.004 |
| Synergistetes[G-3] sp. OT361 II | 0.364 | 0.034 | 0.004 |
| *Prevotella dentalis* | 0.361 | 0.036 | 0.005 |
| TM7[G-1] sp. OT349 | 0.359 | 0.037 | 0.005 |
| *Fusobacterium nucleatum* ss. *vincentii* | 0.357 | 0.038 | 0.005 |
| *Neisseria* sp. (*Neisseria sicca*) | -0.462 | 0.006 | 0.001 |
| *Streptococcus* sp. (*Streptococcus sanguinis*) | -0.454 | 0.007 | 0.001 |
| *Streptococcus gordonii* | -0.44 | 0.009 | 0.002 |
| *Streptococcus sanguis* | -0.44 | 0.009 | 0.001 |
| *Actinomyces* sp. OT169 | -0.432 | 0.011 | 0.002 |
| *Gemella sanguinis* | -0.401 | 0.019 | 0.002 |
| *Streptococcus* sp. OT070 | -0.396 | 0.021 | 0.003 |
| *Rothia aeria* | -0.381 | 0.026 | 0.003 |
| *Actinomyces* sp. (OT170) | -0.37 | 0.031 | 0.004 |
| *Solobacterium moorei* | -0.363 | 0.035 | 0.004 |
| *Streptococcus* sp. (*Streptococcus vestibularis*) | -0.361 | 0.036 | 0.005 |
