# Supplementary material for: Microbiome Profiles in Periodontitis in Relation to Host and Disease Characteristics
Source: PLoS One. 2015 May 18;10(5):e0127077. doi: 10.1371/journal.pone.0127077 (PMC4436126; doi:10.1371/journal.pone.0127077)
Supplement: S5 Table — (DOCX) [file pone.0127077.s012.docx]

**S5 Table**. Spearman Rank Order correlation tests between relative abundances of individual OTUs (top 300 most abundant) and % of sites with PD ≥ 5mm. Only OTUs with *P* values < 0.05 are shown. Bold indicates significant correlations after multiple test adjustment.

| OTU | Correlation coefficient (*r_s_*) | P-value | Q-value |
| --- | --- | --- | --- |
| ***Fusobacterium nucleatum* ss. *vincentii*** | **0.586** | **<0.0001** | **0.0002** |
| *Eubacterium*[11][G-6] *nodatum* | 0.552 | 0.001 | 0.0003 |
| *Treponema* sp. | 0.491 | 0.003 | 0.001 |
| *Tannerella forsythia* | 0.486 | 0.004 | 0.001 |
| *Porphyromonas gingivalis* | 0.475 | 0.004 | 0.001 |
| *Filifactor alocis* | 0.444 | 0.008 | 0.002 |
| *Desulfobulbus* sp. OT041 | 0.440 | 0.009 | 0.002 |
| *Filifactor alocis* | 0.421 | 0.013 | 0.003 |
| *Fusobacterium* sp. (*Fusobacterium nucleatum* ss *vincentii*) | 0.419 | 0.014 | 0.004 |
| *Eubacterium*[11][G-3] *brachy* | 0.400 | 0.019 | 0.004 |
| Peptostreptococcaceae[11][G-4] sp. (OT369) | 0.386 | 0.024 | 0.004 |
| *Dialister pneumosintes* | 0.381 | 0.026 | 0.005 |
| *Prevotella intermedia* | 0.372 | 0.030 | 0.005 |
| *Treponema* sp. | 0.371 | 0.031 | 0.005 |
| *Prevotella* sp. OT473 | 0.365 | 0.034 | 0.006 |
| *Treponema maltophilum* | 0.361 | 0.036 | 0.006 |
| *Treponema* sp. (OT237) | 0.359 | 0.037 | 0.006 |
| *Porphyromonas endodontalis* | 0.351 | 0.042 | 0.007 |
| *Prevotella buccae* | -0.519 | 0.002 | 0.001 |
| *Actinomyces* sp. | -0.503 | 0.002 | 0.001 |
| *Streptococcus sanguis* | -0.462 | 0.006 | 0.001 |
| *Solobacterium moorei* | -0.462 | 0.006 | 0.002 |
| Lachnospiraceae[G-4] sp. (*Moryella* sp. OT419) | -0.456 | 0.007 | 0.002 |
| *Actinomyces* sp. OT169 | -0.444 | 0.008 | 0.002 |
| *Atopobium parvulum* | -0.444 | 0.009 | 0.002 |
| *Actinomyces* sp. (OT896) | -0.437 | 0.010 | 0.003 |
| *Actinomyces* sp. (*Actinomyces odontolyticus*) | -0.428 | 0.011 | 0.003 |
| *Granulicatella adiacens* | -0.422 | 0.013 | 0.003 |
| *Veillonella parvula* | -0.421 | 0.013 | 0.003 |
| *Streptococcus* sp. (*Streptococcus sanguinis*) | -0.417 | 0.014 | 0.003 |
| *Corynebacterium durum* | -0.399 | 0.020 | 0.004 |
| *Prevotella oulorum* | -0.387 | 0.024 | 0.004 |
| *Streptococcus gordonii* | -0.387 | 0.024 | 0.004 |
| *Actinomyces* sp. | -0.382 | 0.026 | 0.005 |
| *Actinomyces naeslundii* | -0.374 | 0.029 | 0.005 |
| *Rothia aeria* | -0.366 | 0.033 | 0.005 |
| *Actinomyces naeslundii* II | -0.363 | 0.035 | 0.006 |
| *Atopobium rimae* | -0.356 | 0.039 | 0.006 |
| *Streptococcus* sp. (*Streptococcus vestibularis*) | -0.352 | 0.041 | 0.006 |
