# Supplementary material for: Microbiome Profiles in Periodontitis in Relation to Host and Disease Characteristics
Source: PLoS One. 2015 May 18;10(5):e0127077. doi: 10.1371/journal.pone.0127077 (PMC4436126; doi:10.1371/journal.pone.0127077)
Supplement: S6 Table — (DOCX) [file pone.0127077.s013.docx]

**S6 Table**. Spearman Rank Order correlation tests between relative abundances of individual OTUs (top 300 most abundant) and PDs of sampled sites. Only OTUs with *P* values < 0.05 are shown. No correlation was significant after multiple test adjustment.

| OTU | Correlation coefficient (*r_s_*) | P-value | Q-value |
| --- | --- | --- | --- |
| *Fusobacterium* sp. (*F. nucleatum* ss *vincentii*) | 0.560 | 0.001 | 0.0003 |
| *Fusobacterium nucleatum* ss. *vincentii* | 0.545 | 0.001 | 0.0002 |
| *Dialister pneumosintes* | 0.523 | 0.002 | 0.001 |
| *Eubacterium*[11][G-3] *brachy* | 0.505 | 0.002 | 0.001 |
| Veillonellaceae[G-1] sp. OT155 | 0.490 | 0.003 | 0.001 |
| *Neisseria* sp. (*Neisseria flavescens*) | 0.481 | 0.004 | 0.001 |
| *Gemella* sp. (*Gemella haemolysans*) | 0.458 | 0.006 | 0.001 |
| *Tannerella forsythia* | 0.450 | 0.008 | 0.002 |
| *Treponema* sp. | 0.449 | 0.008 | 0.002 |
| *Eubacterium*[11][G-6] *nodatum* | 0.444 | 0.009 | 0.002 |
| *Filifactor alocis* | 0.444 | 0.009 | 0.002 |
| *Prevotella intermedia* | 0.436 | 0.010 | 0.003 |
| *Campylobacter* sp. OT044 | 0.435 | 0.010 | 0.003 |
| *Prevotella* sp. OT473 | 0.416 | 0.014 | 0.003 |
| *Filifactor alocis* | 0.409 | 0.016 | 0.003 |
| TM7[G-1] sp. OT349 | 0.396 | 0.021 | 0.004 |
| *Cardiobacterium hominis* | 0.393 | 0.022 | 0.004 |
| *Kingella denitrificans* | 0.388 | 0.023 | 0.004 |
| Peptostreptococcaceae[11][G-4] sp. (OT369) | 0.386 | 0.024 | 0.004 |
| *Haemophilus* sp. (OT908) | 0.373 | 0.030 | 0.005 |
| *Fusobacterium* sp. (*F. nucleatum* ss. *nucleatum*) | 0.367 | 0.033 | 0.005 |
| *Leptotrichia* sp. OT212 | 0.353 | 0.040 | 0.005 |
| *Prevotella* sp. (*Alloprevotella* sp. OT912) | 0.349 | 0.043 | 0.005 |
| *Actinomyces* sp. (OT896) | -0.465 | 0.006 | 0.001 |
| *Prevotella buccae* | -0.447 | 0.008 | 0.002 |
| *Streptococcus sanguis* | -0.439 | 0.009 | 0.002 |
| *Granulicatella adiacens* | -0.429 | 0.011 | 0.003 |
| *Actinomyces naeslundii* | -0.409 | 0.016 | 0.003 |
| *Atopobium parvulum* | -0.404 | 0.018 | 0.004 |
| *Actinomyces naeslundii* II | -0.387 | 0.024 | 0.004 |
| *Actinomyces* sp. | -0.365 | 0.034 | 0.005 |
| *Streptococcus gordonii* | -0.352 | 0.041 | 0.005 |
