# Supplementary material for: Microbiome Profiles in Periodontitis in Relation to Host and Disease Characteristics
Source: PLoS One. 2015 May 18;10(5):e0127077. doi: 10.1371/journal.pone.0127077 (PMC4436126; doi:10.1371/journal.pone.0127077)
Supplement: S1 Supporting Methods — (PDF) [file pone.0127077.s014.pdf]

Supporting Methods

Comparison of healthy subjects from the Human Microbiome Project (HMP) cohort to periodontitis subjects from the current study

Data from periodontally healthy subjects, publicly available via the HMP [1], was used to confirm associations of different taxa with health or periodontitis. According to the HMP Manual of Procedures (http://hmpdacc.org/doc/HMP_MOP_Version12_0_072910.pdf), 18 to 40 year old subjects were enrolled at two US locations and did not have 1) PDs ≥ than 4 mm; 2) > 8 missing teeth and 3) > 10% of sites with BoP. Moreover, among inclusion criteria were no antibiotic use within six months and no chronic medical conditions. It is reported healthy subjects had subgingival plaque collected from the mesio buccal surface of six index teeth, prior to pooling of same subject samples.

Sff files from all subjects with sequenced V1-V3 regions of the 16S rRNA gene were downloaded from the HMP website (http://www.hmpdacc.org/HMR16S/). HMP amplicons had been generated using primers 27F 5'AGAGTTTGATCCTGGCTCAG3' and 534R 5'ATTACCGCGGCTGCTGG3' and sequenced in the reverse direction on the 454 platform. DNA isolation procedures, library construction and sequencing protocols were accessed at http://www.hmpdacc.org/doc/HMP_MDG_454_16S_Protocol.pdf.

Raw data files from the HMP and periodontitis datasets were processed simultaneously in Mothur [2, 3]. Briefly, datasets were denoised, trimmed, and chimeras removed. Since datasets were sequenced from opposite directions, only 41 bps overlapped after alignment of trimmed sequences. Therefore, we did not perform an Operational Taxonomic Unit

(OTU)-based analysis to compare the HMP and periodontitis datasets, but followed the "phylotype pipeline" described by Schloss *et al.* [3]. This involves binning of sequences according to taxonomy, which was determined using Mothur's version of the Ribosomal Database Project (RDP) classifier [4] and the HOMD [5] as template.

HMP and periodontitis libraries were randomly subsampled to contain 3,500 sequences. This reduced the dataset to 79 healthy subjects and 34 with periodontitis. The Jaccard Index was used to compare distance among communities according to taxa prevalence and the θ_{YC} index [6] to estimate distances according to structure. Principal Coordinate Analysis (PCoA) of distances among communities based on these metrics was performed in Mothur and graphs visualized using the rgl application within R (http://www.r-project.org/). Amova [7] was used to test if separation between health and periodontitis data clouds was significant. Differences in phylotype abundances between health and periodontitis were determined with LEfSe [8] and prevalence differences via χ^2.

β-diversity analysis of periodontitis samples in relation to CKD and diabetes

The Jaccard Index was used to compare distance among periodontitis communities according to OTU prevalence and the θ_{YC} index [6] to estimate distances according to structure. Principal Coordinate Analysis (PCoA) was performed in Mothur and graphs visualized using the rgl application within R (http://www.r-project.org/). Amova [7] was used to test if separation between data clouds was significant.

References cited in this section

1. Human Microbiome Project Consortium: A framework for human microbiome research. Nature 2012; 486(7402): 215-21.

2. Schloss PD, Westcott SL, Ryabin T, Hall JR, Hartmann M, Hollister EB, et al.: Introducing mothur: open-source, platform-independent, community-supported software for describing and comparing microbial communities. Appl Environ Microbiol 2009; 75(23): 7537-41.

3. Schloss PD, Gevers D, and Westcott SL: Reducing the effects of PCR amplification and sequencing artifacts on 16S rRNA-based studies. PLoS One 2011; 6(12): e27310.

4. Wang Q, Garrity GM, Tiedje JM, and Cole JR: Naive Bayesian classifier for rapid assignment of rRNA sequences into the new bacterial taxonomy. Appl Environ Microbiol 2007; 73(16): 5261-7.

5. Dewhirst FE, Chen T, Izard J, Paster BJ, Tanner AC, Yu WH, et al.: The human oral microbiome. J Bacteriol 2010; 192(19): 5002-17.

6. Yue JC and Clayton MK: A similarity measure based on species proportions. Communications in Statistics Theory and Methods 2005; 34: 2123-2131.

7. Excoffier L, Smouse PE, and Quattro JM: Analysis of molecular variance inferred from metric distances among DNA haplotypes: application to human mitochondrial DNA restriction data. Genetics 1992; 131(2): 479-91.

8. Segata N, Izard J, Waldron L, Gevers D, Miropolsky L, Garrett WS et al.: Metagenomic biomarker discovery and explanation. Genome Biol 2011; 12(6): R60.
